# Consensus on Recommended Functions of a Smart Home System to Improve Self-Management Behaviors in People With Heart Failure: A Modified Delphi Approach

**DOI:** 10.3389/fcvm.2022.896249

**Published:** 2022-06-29

**Authors:** Sheikh Mohammed Shariful Islam, Rebecca Nourse, Riaz Uddin, Jonathan C. Rawstorn, Ralph Maddison

**Affiliations:** Institute for Physical Activity and Nutrition, Deakin University, Geelong, VIC, Australia

**Keywords:** Delphi survey, cardiovascular diseases, lifestyle behaviors, self care, health monitoring, information technology

## Abstract

**Background:**

Smart home systems could enhance clinical and self-management of chronic heart failure by supporting health monitoring and remote support, but evidence to guide the design of smart home system functionalities is lacking.

**Objective:**

To identify consensus-based recommendations for functions of a smart home system that could augment clinical and self-management for people living with chronic heart failure in the community.

**Methods:**

Healthcare professionals caring for people living with chronic heart failure participated in a two-round modified Delphi survey and a consensus workshop. Thirty survey items spanning eight chronic health failure categories were derived from international guidelines for the management of heart failure. In survey Round 1, participants rated the importance of all items using a 9-point Liket scale and suggested new functions to support people with chronic heart failure in their homes using a smart home system. The Likert scale scores ranged from 0 (not important) to 9 (very important) and scores were categorized into three groups: 1–3 = not important, 4–6 = important, and 7–9 = very important. Consensus agreement was defined a priori as ≥70% of respondents rating a score of ≥7 and ≤ 15% rating a score ≤ 3. In survey Round 2, panel members re-rated items where consensus was not reached, and rated the new items proposed in earlier round. Panel members were invited to an online consensus workshop to discuss items that had not reached consensus after Round 2 and agree on a set of recommendations for a smart home system.

**Results:**

In Round 1, 15 experts agreed 24/30 items were “very important”, and suggested six new items. In Round 2, experts agreed 2/6 original items and 6/6 new items were “very important”. During the consensus workshop, experts endorsed 2/4 remaining items. Finally, the expert panel recommended 34 items as “very important” for a smart home system including, healthy eating, body weight and fluid intake, physical activity and sedentary behavior, heart failure symptoms, tobacco cessation and alcohol reduction, medication adherence, physiological monitoring, interaction with healthcare professionals, and mental health among others.

**Conclusion:**

A panel of healthcare professional experts recommended 34-item core functions in smart home systems designed to support people with chronic heart failure for self-management and clinical support. Results of this study will help researchers to co-design and protyping solutions with consumers and healthcare providers to achieve these core functions to improve self-management and clinical outcomes in people with chronic heart failure.

## Key Points

- **Question:** What essential functions are recommended by healthcare professionals for a Smart Home system for people with heart failure?
- **Findings:** An expert panel of healthcare professionals agreed on 34 items as essential functions for a smart home system to support self-care for heart failure, including healthy eating, body weight and fluid intake, physical activity and sedentary behavior, monitoring of symptoms, tobacco cessation and alcohol reduction, medication adherence, physiological monitoring, interaction with healthcare professionals, and mental health.
- **Meaning:** The recommendations from the expert panel can guide the development of future smart home systems for people with heart failure.

## Introduction

Chronic heart failure is an increasingly prevalent condition and is associated with a considerable health burden (1). Despite significant advances in medical treatment, approximately 44% of people with heart failure are re-hospitalized within 1 year of discharge (2), and 50% die within 5 years (3). This is primarily due to rapid health deterioration, severe comorbidities and lack of post-acute care monitoring. International guidelines recommend self-management as an essential strategy to improve care for people with chronic heart failure (4, 5). Self-management includes monitoring symptoms, adhering to prescribed medications, and adopting and maintaining lifestyle behaviors such as a healthy diet and physical activity (5, 6). A meta-analysis of patient-level data from 20 trials (*n* = 5,624 patients) demonstrated that self-management interventions reduced the risk of time to the combined endpoint of heart failure-related hospitalization or all-cause death (HR 0.80; 95% CI 0.71–0.89), and time to heart failure-related hospitalization (HR 0.80; 0.69–0.92) (7). However, sub-optimal symptom recognition, a lack of patient education, delayed symptom reporting, and medication non-adherence make optimal self-management challenging (8, 9). Innovative approaches that support people to better manage their heart failure are needed to improve individual's health and wellbeing, and ease burden on the healthcare system.

Previous trials involving implantable devices, mobile phone applications, text messaging, web-based programs and telemonitoring have shown to support self-management in people with heart failure (10–12). A systematic review and meta-analysis of randomized controlled trials, comparing whether people with heart failure received telemedicine or usual standard care, showed overall all-cause mortality (pooled OR = 0.80, 0.71 to 0.91, *p* < 0.001) and heart failure-related admission rate (pooled OR = 0.63, 0.53 to 0.76, *p* < 0.001) were significantly lower in the telemedicine group (13). Notwithstanding these findings, telemedicine has focussed predominantly on medical outcomes (such as symptoms) and has largely ignored the need to support people with heart failure to better self-manage their condition. Technology has the potential to address these limitations, by empowering people with heart failure to better manage their health, and to optimize communication with their clinicians when and if needed. Further, current approaches for designing technology interventions have typically failed to include patients and clinicians in the product design (14), which leads to lower levels of acceptance, dissatisfaction, stress and non-adherence (15–17).

**Figure 1:**
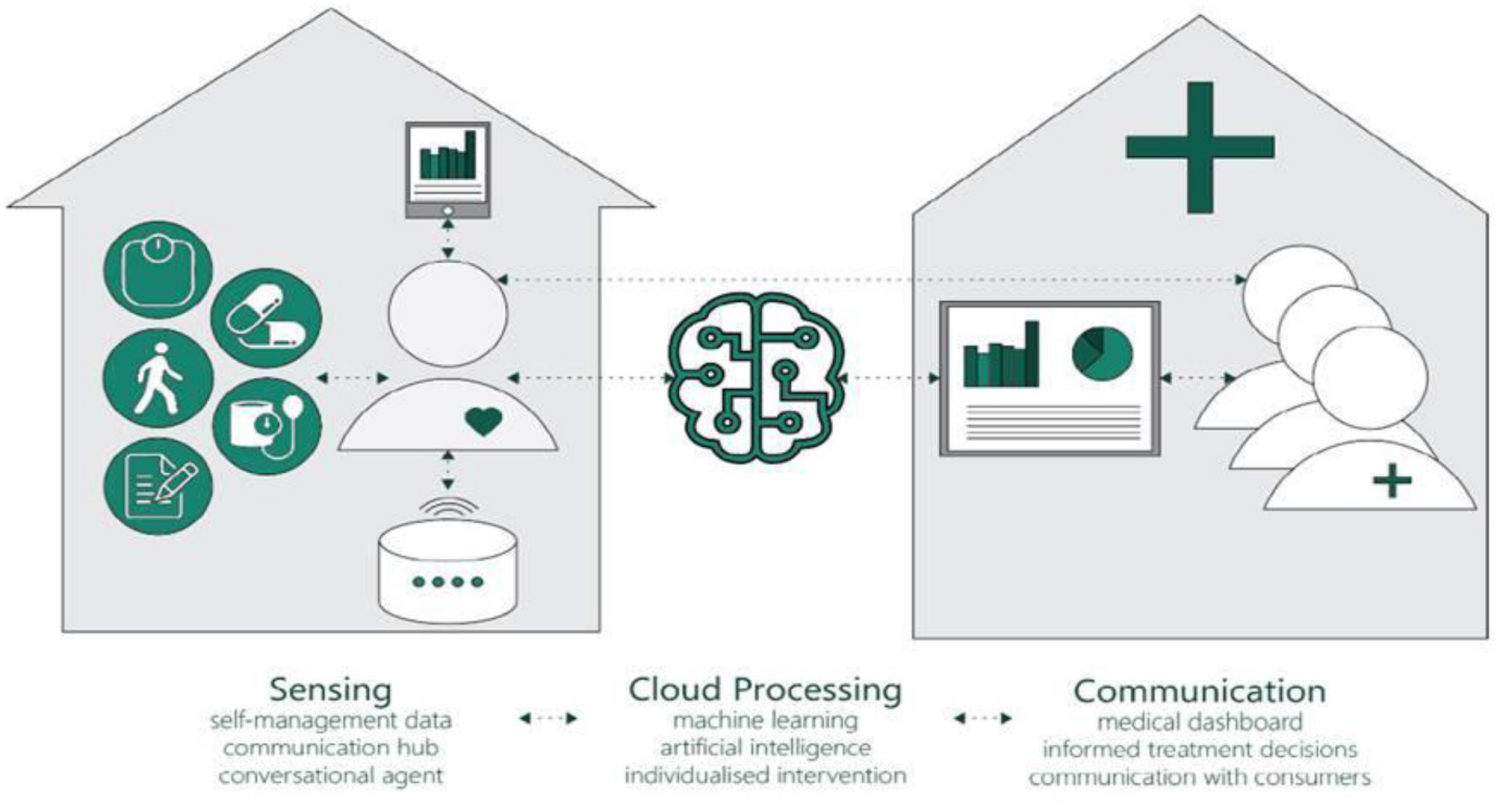
A conceptual framework of Smart Home for people with heart failure.

In recent years, more smart technology solutions, which move beyond soley monitoring have emerged. Smart solutions incorporate network-connected sensors and communication platforms, have been used to monitor people's daily activities, communicate with care providers and support independent living (18–20). These smart systems have the potential to enable post-discharge monitoring, detect a worsening health status, allow healthcare professionals to tailor treatments remotely and support people to be proactive in seeking support (21). In collaboration with clinical, behavioral, and information technology experts, we are developing a smart home system for people living with chronic heart failure in the community. The smart home system connects different elements to support self-management of people living with chronic heart failure, thereby improving health ouctomes. Smart home systems incorporate network-connected sensors and communication platforms and have been used in recent years to monitor patients' daily activities, communicate with care providers, and support independent living (18, 19, 22, 23). For the purpose of this research, a smart home ecosystem carries out three key actions: sensing, processing, and communication (24). Specifically, the system will connect sensors (e.g., wearable and environmental) and medical devices (e.g., blood pressure monitor), send data from these devices to a cloud-based server for interpretation, and provide feedback to the end-users (people with chronic heart failure and healthcare professionals) (Figure 1). The system will also facilitate communication between people living with chronic heart failure and healthcare professionals. One step in the development process is to identify necessary functions. To the best of our knowledge, no published studies have undertaken this work. Therefore, we aimed to develop a consensus-based set of core functions, based on international guidelines, that healthcare professionals recommend for inclusion in a smart home system to support people living with chronic heart failure in their homes.

## Methods

We used a modified Delphi survey methodology with two survey rounds and an online consensus workshop. The Delphi technique is a *well*-established approach to answering a research question through the identification of a consensus view across subject experts. This study is compliant with the “Recommendations for the Conducting and REporting of DElphi Studies” (CREDES) (25). Delphi consensus processes systematically aggregate expert input to identify areas of agreement and are commonly used to develop clinical guidelines, standards and quality measures(26). Consensus methods are appropriate where published information is inadequate or non-existent to provide a means of harnessing the insights of appropriate experts to enable decision making (27). Furthermore, the anonymous and iterative features of the Delphi method permit panel members to share their opinion without any individual dominance and peer pressure, which offers an advantage over other group research methods (28).

### Participants and Panel Recruitment

Panel members with experience caring for people with chronic heart failure were sought from a range of healthcare professionals (e.g., general practitioners, cardiologists, nurses, pharmacists, and physiotherapists) from clinical and academic settings. A research team member (SMSI) established initial contact with potential panel members *via* email, phone, or an in-person meeting. Contacts were also asked whether they could recommend others who may add value to the project. The final selection of panel members aimed to ensure representation from multiple clinical fields. The identity of panel members was kept confidential throughout the survey process to ensure that each member felt free to agree or disagree with other members' responses.

### Delphi Surveys

A limit of two survey rounds was chosen to reduce the panel members' burden and ensure a high response rate. Each survey was tested prior to distribution using people who were not participants in the Delphi rounds. They were asked to consider comprehension and the structure and readability of statements, and to identify any procedural problems when administering the surveys. Round 1 was tested by five clinical researchers, including a general practitioner, cardiologist, nurse, pharmacist, and physiotherapist with clinical experience in managing patients with heart failure for completeness, applicability and clarity. Round 2 was tested by three clinician researchers. Panel members received links to electronic surveys (hosted on Qualtrics) via email, and were asked to complete each survey within 2 weeks, with reminders sent after 1 week. Panel members rated the importance on a 9-point Likert scale ranging from 0 (not important) to 9 (very important) and scores were categorized into three groups: not important (score 1 to 3), important (score 4 to 6), and very important (score 7 to 9). The scale and the scoring were based on the Grading of Recommendations Assessment, Development and Evaluation (GRADE) approach, which is used to rate the strength and quality of evidence (29). Instructions to panel members were to consider the importance of including each item in a smart home system for delivering information technology-supported care at home to a typical patient with chronic heart failure living in the community. Furthermore, panel members were instructed not to consider cost implications when making judgements to ensure ratings were not impacted by a lack of information about the technologies and costs needed to deliver each component. Participant anonymity was maintained by individualized communication for each round.

### Survey Round 1

Survey items were derived from a review of the literature and relevent guidelines for people with chronic heart failure. Sources included international heart failure guidelines (4, 5, 30, 31) and previous systematic reviews (1, 8). Given the lack of published research on the topic, a free-text item asked panel members to suggest other items that they felt may warrant inclusion in a smart home system. The first survey consisted of 30 items grouped into eight categories (Table 1): (1) healthy eating, body weight and fluid intake; (2) physical activity and sedentary behavior; (3) heart failure symptoms; (4) tobacco cessation and alcohol reduction; (5) medication adherence; (6) physiological monitoring; (7) interaction with healthcare professionals; and (8) mental health.

**Table 1:** Delphi survey round 1 scoring and classification.

**Item serial number/ Items**	**Median**	**% rated ≥7**	**% rated ≤3**	**Consensus**	**Classification**
**Healthy eating, body weight and fluid intake**
*A Smart Home should provide education on…*					
1. healthy dietary choices (e.g., fresh fruits/vegetables)	8	80	7	Yes	Very important
2. low sodium diet	8	80	0	Yes	Very important
3. weight monitoring	9	93	0	Yes	Very important
4. fluid monitoring	8	87	0	Yes	Very important
**Physical activity and sedentary behavior**
*A Smart Home should…*					
5. provide individualized exercise prescription	8	80	0	Yes	Very important
6. monitor physical activity behaviors	8	87	0	Yes	Very important
7. monitor sedentary behaviors	8	73	0	Yes	Very important
**Heart failure symptoms**
*A Smart Home should…*					
8. help participants to record heart failure symptoms (e.g., shortness of breath, swelling of legs, fatigue, and weakness)	8	87	0	Yes	Very important
9. remind participants about monitoring their heart failure symptoms	8	80	0	Yes	Very important
10. engage individuals to monitor their symptoms	8	73	0	Yes	Very important
**Tobacco cessation and alcohol reduction**
*A Smart Home should…*					
11. provide support to reduce/quit tobacco	8	93	0	Yes	Very important
12. provide support to reduce/quit alcohol consumption	8	93	0	Yes	Very important
**Medication adherence**
*A Smart Home should…*					
13. provide information on medication adherence	8	87	0	Yes	Very important
14. provide medication alerts (e.g., sensors on medication packs)	8	87	0	Yes	Very important
15. provide reminders to take prescribed medications	9	100	0	Yes	Very important
**Physiological monitoring**
*A Smart Home should…*					
16. monitor blood pressure	9	100	0	Yes	Very important
17. monitor heart rate	9	93	0	Yes	Very important
18. monitor blood glucose	8	60	0	No	Round 2
19. monitor sleep duration	7	53	0	No	Round 2
20. monitor weight	9	93	0	Yes	Very important
21. monitor fluid intake	8	67	7	No	Round 2
22. monitor medication use	8	100	0	Yes	Very important
23. monitor diet	7	67	0	No	Round 2
**Interaction with healthcare professionals**
*A Smart Home should …*					
24. support communication between users and healthcare providers	8	93	7	Yes	Very important
25. be linked with clinical management systems (e.g., electronic health records)	8	93	0	Yes	Very important
26. provide reminders for clinic appointments	8	87	0	Yes	Very important
27. provide alerts to healthcare providers about the patient's deteriorating condition	9	93	0	Yes	Very important
**Mental health**
*A Smart Home should…*					
28. monitor individuals' mental health (e.g., depressive symptoms)	8	80	0	Yes	Very important
29. connect with a mental health support system (e.g., Beyond Blue)	8	67	0	No	Round 2
30. provide support for optimizing mental health in the form of messages delivered via a conversational agent (e.g., Google Home, Alexa)	7	53	0	No	Round 2

*Consensus for an item was defined as: ≥ 70% of respondents rated a score ≥7 and ≤ 15% of respondents rated a score ≤ 3 [Likert scale: 1 (not important) to 9 (very important)]*.

### Survey Round 2

Round 2 was developed based on Round 1 analysis. Panel members were invited to re-rate items where consensus agreement was not reached in the previous round and rate new items generated from free-text responses in Round 1.

### Consensus Workshop

Panel members were invited to an online consensus workshop by email, and the research team followed up with panel members who did not respond to the initial email. The workshop began with a welcome and introduction by SMSI, followed by a review of the workshop objectives, an agenda, and information about the group activity and introductions of the research team and panel members. To provide additional context for the discussion, researchers with experience in digital health for chronic disease management presented their findings related to a scoping review of smart home-based systems for chronic disease management, a conceptual framework for a smart home ecosystem (Figure 1) and the preceding Delphi surveys. The workshop facilitator (RN) then encouraged the panel members to generate reasons to accept or reject survey items that did not reach consensus, promote discussion and guide panel members to reach a consensus agreement for each item.

### Data Analysis

Participant characteristics and survey response rates are reported descriptively. For Likert scale items, the median rating, % rated ≥ 7 and % rated ≤ 3 were calculated. Consensus for both rounds was defined as follows; items were classified as important if ≥70% of respondents rated a score ≥7 and ≤ 15% of respondents rated a score ≤ 3 (32). During the workshop, items were categorized as either “endorsed” if participant responses were positive (i.e., use of words like “agree,” “support,” “good”). The final list of recommended functions included items that reached consensus agreement as “important” or were endorsed during the workshop.

### Ethics Approval

The study was approved by Deakin University Human Research Ethics Committee (HEAG-H 151_2019). All participants provided informed consent.

## Results

In total, 21 experts were invited to participate in the study; 15 provided consent and formed the panel (*n* = 9 female, *n* = 4 general practitioner (GP), *n* = 3 cardiologist, *n* = 3 physiologist, *n* = 2 pharmacist, *n* = 2 nurse, and *n* = 1 dietician). All participants responded to both surveys. Only four panel members were able to attend the workshop (*n* = 2 GP, *n* = 1 cardiologist, and *n* = 1 nurse) therefore, a short report outlining the results was circulated by email to the panel with a request for feedback arising from any concerns; no panel members responded with disagreements.

### Survey Round 1

Consensus was reached for 24 of 30 items (80%); all were rated as “very important” (Table 1). No items were considered “not important”. Items that did not achieve consensus related to physiological monitoring (items 18, 19, 21, and 23) and mental health (items 29 and 30). Seven experts provided optional free-text responses, and six additional items were generated from these responses (items A1 to A6).

### Survey Round 2

**Figure 2:**
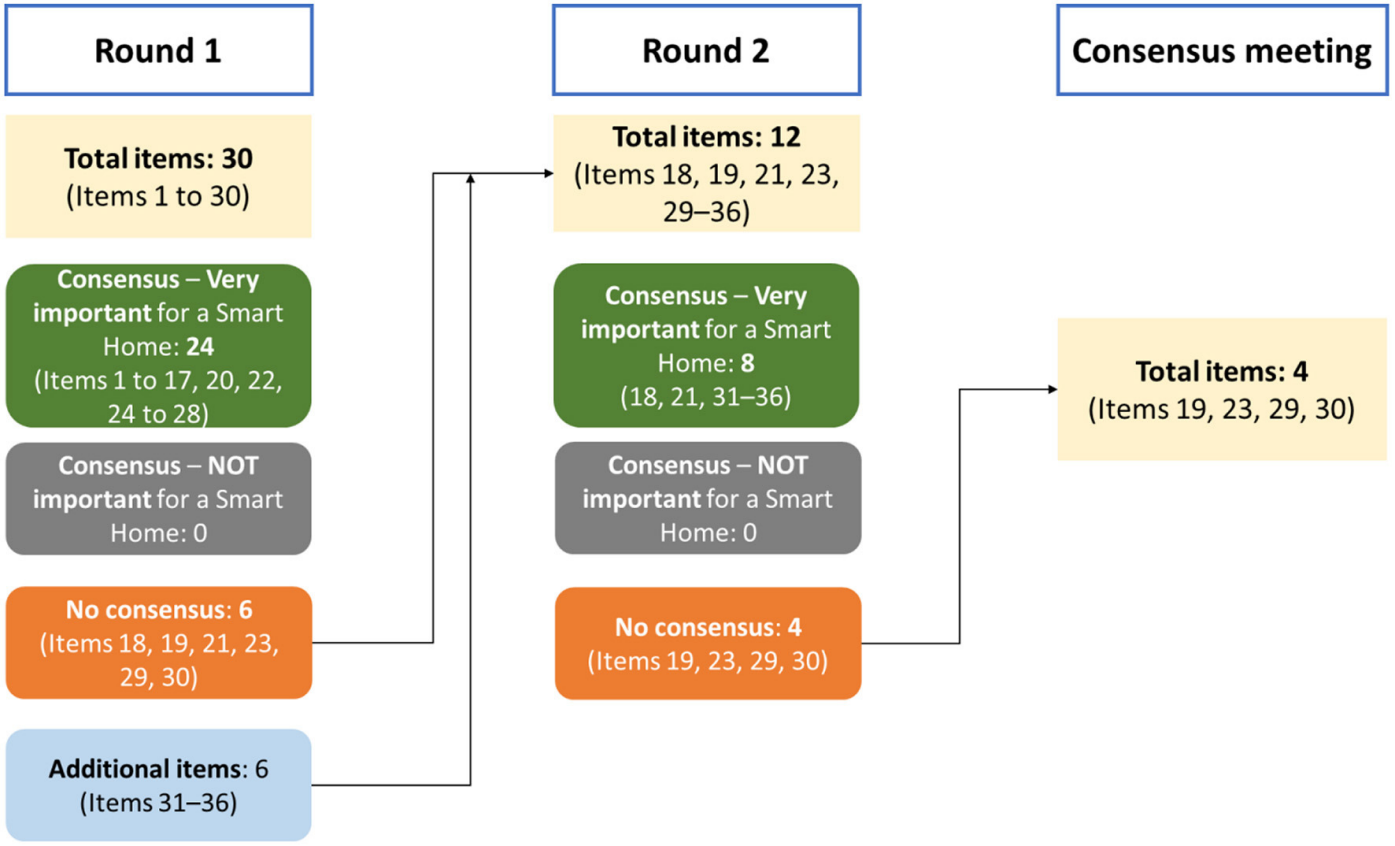
Study flowchart.

The second survey (Table 2) consisted of six items where no consensus was reached (18, 19, 21, 23, 29, 30) and six new items suggested by the panel during Round 1 (31–36). Items 18 and 21 reached consensus agreement as “very important”. Items related to physiological monitoring (19 and 23) and mental health (29 and 30) (Figure 2). All new items suggested by the panel in Round 1 reached a consensus agreement as “very important”.

**Table 2:** Delphi survey round 2 scoring and classification.

**Item serial number/ Items**	**Median**	**% rated ≥7**	**% rated ≤3**	**Consensus**	**Classification**
**Items from round 1**	
*A Smart Home should …*					
18. monitor blood glucose	8	73	13	Yes	Very important
19. monitor sleep duration	7	**67**	7	**No**	Discuss
21. monitor fluid intake	8	80	0	Yes	Very important
23. monitor diet	7	**67**	0	**No**	Discuss
29. connect with a mental health support system (e.g., Beyond Blue)	7	**60**	7	**No**	Discuss
30. provide support for optimizing mental health in the form of messages delivered via a conversational agent (e.g., Google Home, Alexa)	6	**40**	0	**No**	Discuss
**Additional items**	
*A Smart Home should…*					
31. monitor ECG	7	73	7	Yes	Very important
32. be able to provide individualized care package (e.g., fluid monitoring in those with multiple fluid overload admission)	8	93	7	Yes	Very important
33. provide peer support (e.g., connect with family, friends)	8	80	0	Yes	Very important
34. use accelerometry for falls monitoring	8	73	0	Yes	Very important
35. remind participants for self-management of medication titration if symptomatic	9	100	7	Yes	Very important
36. have exercise charts and dietary plans	7	73	0	Yes	Very important

*Consensus for an item was defined as: ≥ 70% of respondents rated a score ≥7and ≤ 15% of respondents rated a score ≤ 3 [Likert scale: 1 (not important) to 9 (very important)]*.

### Consensus Workshop

Four items where no consensus was reached during the survey rounds were discussed at the workshop. Discussion of item 23 (“monitoring diet”) centered on feasibility, and the item was subsequently endorsed. Discussion of item 30 (provide support for optimizing mental health in the form of messages delivered via a conversational agent) highlighted a need to ensure messaging interventions to support mental health are personalized. The item was reworded as 'provide personalized mental health messages delivered *via* a conversational agent (e.g., Google Home, Alexa) before being endorsed. Item 19 (“monitor sleep duration” was not endorsed as the panel perceived technical challenges with sleep measurement, and cited a lack of evidence suggesting sleep monitoring affects the clinical presentation of heart failure. Finally, item 29 (“connect with a mental health support system”) was not endorsed as the panel perceived referral to mental health support services would not provide a personalized approach required by people with heart failure.

After two survey rounds and one workshop, 34 items were classified as very important or endorsed by the panel (Box 1).

**Box 1:** Smart Home system functions recommended as “Very important” by the panel.

**Healthy eating, body weight and fluid intake**
1. Provide education on healthy dietary choices (e.g., fresh fruits/vegetables)
2. Provide education on low sodium diet
3. Provide education on weight monitoring
4. Provide education on fluid monitoring
**Physical activity and sedentary behavior**
5. Provide individualized exercise prescription
6. Monitor physical activity behaviors
7. Monitor sedentary behaviors
**Heart failure symptoms**
8. Help participants to record heart failure symptoms (e.g., shortness of breath, swelling of legs, fatigue, weakness)
9. Remind participants about monitoring their heart failure symptoms
10. Engage individuals to monitor their symptoms
**Tobacco cessation and alcohol reduction**
11. Provide support to reduce/quit tobacco
12. Provide support to reduce/quit alcohol consumption
**Medication adherence**
13. Provide information on medication adherence
14. Provide medication alerts (e.g. sensors on medication packs)
15. Provide reminders to take prescribed medications
**Physiological monitoring**
16. Monitor blood pressure
17. Monitor heart rate
18. Monitor blood glucose
20. Monitor weight
21. Monitor fluid intake
22. Monitor medication use
23. Monitor diet
**Interaction with healthcare professionals**
24. Support communication between users and healthcare providers
25. Be linked with clinical management systems (e.g. electronic health records)
26. Provide reminders for clinic appointments
27. Provide alerts to healthcare providers about the patient's deteriorating condition
**Mental health**
28. Monitor individuals' mental health (e.g. depressive symptoms)
30. Provide personalized mental health messages delivered via a conversational agent (e.g., Google Home, Alexa)
**Additional items**
31. Monitor ECG
32. Be able to provide individualized care package (e.g. fluid monitoring in those with multiple fluid overload admission)
33. Provide peer support (e.g. connect with family, friends)
34. Use accelerometry for falls monitoring
35. Remind participants for self-management of medication titration if symptomatic
36. Have exercise charts and dietary plans

## Discussion

To our knowledge, this is the first study to use the Delphi consensus process to identify recommended core functions for smart home systems designed to support people with chronic heart failure living in the community. An experienced multidisciplinary expert panel agreed on 34 core functions related to healthy eating, body weight and fluid intake; physical activity and sedentary behavior; heart failure symptoms monitoring; tobacco cessation and alcohol reduction; medication adherence; physiological monitoring; interaction with healthcare professionals; and mental health among others to be included in smart home systems.

Both survey rounds demonstrated high levels of agreement amongst panel members, with no apparent differences between disciplinary or clinical backgrounds. However, additional clarification was still required on four items in the categories of “physiological monitoring” and “mental health” after the two survey rounds. The workshop provided this clarification and panel members emphasized smart home system monitoring functions should be limited to health parameters that have been found to improve heart failure symptoms and self-management. While monitoring self-management behaviors (physical activity, sedentariness, diet, fluid intake, and medication use), vital signs (blood pressure, ECG/heart rate), and other health parameters (blood glucose concentration, body weight, mental health) were recommended, but monitoring sleep duration was not recommended as a perceived lack of evidence about clinical utility suggesting experts believed sleep data would not enhance clinical management.

Experts specified that messaging to support mental health should be personalized. A similar approach may be important for other smart home functions as personalized interventions have been shown to improve behavior change and maintenance (33). However, persaonlization was not explicitly raised by the panel in relation to other functions. Recommended monitoring functions could play key roles in personalizing smart home system functionality by enabling a better understanding of individuals' self-management behaviors and health status. These data could also be shared with clinicians to enable more personalized clinical management; however, consideration is needed on how to achieve this without overwhelming healthcare professionals. Smart home systems can provide personalization at different levels, for example functions can be easily added or in their entirety, or tailored in their execution by applying data analytics to sensed data. This is pertinent to people living with chronic heart failure who may have different needs (e.g., types of support at different stages of the disease trajectory) and preferences (e.g., willingness to use different types of digital technologies).

This study is an important contribution to the literature. Delphi processes for establishing expert consensus have been used to develop clinical guidelines, quality measures and identify important processes of care associated with heart failure (26, 34). We extended these methods to the realm of a smart home system for people with heart failure. The Delphi process allowed for data to be collected anonymously, systematically and iteratively which allowed for reasoned expert feedback with less bias from more forthright participants (35). Whilst we recruited a multidisciplinary panel of experts from a range of clinical backgrounds, it was not representative of all clinicians involved in caring for people with heart failure. Other clinicians may have arrived at different conclusions with more opposing views and debate resulting in a longer and more challenging process of achieving consensus. Therefore, our findings should be considered as an initial step in establishing the core functions of a smart home system for supporting self-management and clinical management in chronic heart failure (27).

While there was a high level of agreement between experts, the results should be interpreted with the following limitations: First, the homogenous scores across most items made it problematic to determine their importance relative to one another. Second, the small sample size (n=15) could be identified as a limitation. However, there is no standard method to calculate the number of experts required for a Delphi study, which can range from a few to hundreds of participants (36), depending on the study objectives, group heterogeneity, and available resources (36, 37). A heterogeneous group of 5–30 experts have been suggested to reduce bias in opinion, given that increasing the sample size does not result in improved outcomes and can reduce the response rate (36–38). Whilst all panel members responded to both survey rounds, only four attended the workshop which is lower than suggested previously (36–38). However, this smaller group size did allow for all voices to be heard in the workshop discussion, and different clinical backgrounds still represented a range of expertise. Third, the consensus workshop took place 12-months after the final survey due to the challenges associated with the COVID-19 pandemic (e.g., additional clinical load), this may have contributed to the low workshop participation and concerns that panel members would not remember details of the surveys. The workshop presentations aimed to resolve this, re-orienting the panel to the research topic and the survey items. Finally, as panel members were asked not to consider cost, these recommendations should be viewed as a guide for ideal functionality. If resource constraints prevent execution of all functions, additional work may be needed to inform an iterative development roadmap that prioritizes functions based anticipated benefits and costs.

This study contributes a significant element toward the development of a smart home system to support clinicial and self-management in people with chronic heart failure. A smart home system with these functions could contribute to evidence gaps outlined in international clinical guidelines, including the need for more data on the effects of fluid restriction, dietary salt restriction and nutrition; the role of remote monitoring; optimal models for follow-up of stable heart failure patients; better definition and classification of patient phenotypes to facilitate improved treatment; and development of better strategies for congestion relief, including monitoring of diuretic administration (5, 6). Smart homes can address these gaps by collecting these data directly from patients' home, using machine learning algorithms to create phenotypes, providing automated alerts, remote medication titrations and care (39–41). The findings may also have implications for technology-based programs for other chronic diseases in which self-management is important (e.g., chronic obstructive pulmonary disease). However, further work is necessary to determine the condition-specific functions, test specific items to determine the practical feasibility, usability and validity of this approach and frameworks (42–44). Combining the results of this study with findings from formative research that gathers user insights will guide the development of an innovative, intelligent smart home system for people with chronic heart failure. Furthermore, work to determine the most useful functions for different disease phenotypes and people at different points on the disease trajectory (e.g., acute or chronic decompensation, de novo or worsening patients, patients with preserved or reduced ejection fraction) is needed (45). Once functional prototypes have been developed, clinical trials will be needed to understand the effectiveness and cost-effectiveness of smart home systems in improving health outcomes in people with chronic heart failure.

## Conclusion

A multidisciplinary panel of heart failure clinicians recommended 34 functions spanning healthy eating, body weight and fluid intake, physical activity and sedentary behavior, heart failure symptoms, tobacco cessation and alcohol reduction, medication adherence, physiological monitoring, interaction with healthcare professionals, and mental health among others for inclusion in smart home systems designed to enhance clinical and self-management of chronic heart failure. Results of this study will help researchers to co-design and protyping solutions with consumers and healthcare providers to achieve these core functions to improve self-management and clinical outcomes in people with chronic heart failure.

## Data Availability Statement

The raw data supporting the conclusions of this article will be made available by the authors, without undue reservation.

## Ethics Statement

The studies involving human participants were reviewed and approved by the Deakin University Human Research Ethics Committee. The patients/participants provided their written informed consent to participate in this study.

## Author Contributions

Full access to all of the data in the study and take responsibility for the integrity of the data and the accuracy of the data analysis: SMSI. Concept and design and study supervision: SMSI and RM. Acquisition, analysis, or interpretation of data: SMSI and RU. Drafting of the manuscript and administrative, technical, or material support: SMSI, RN, RU, JR, and RM. Statistical analysis: RU. Obtained funding: SMSI. Critical revision of the manuscript for important intellectual content: All authors. All authors contributed to the article and approved the submitted version.

## Funding

SMSI is funded by the National Heart Foundation of Australia (102112) and a National Health and Medical Research Council (NHMRC) Emerging Leadership Fellowship (APP1195406). RU is supported by Alfred Deakin Postdoctoral Research Fellowship. The funder had no role in the design and conduct of the study, collection, management, analysis, interpretation of the data, preparation, review, or approval of the manuscript, and decision to submit the manuscript for publication.

## Conflict of Interest

The authors declare that the research was conducted in the absence of any commercial or financial relationships that could be construed as a potential conflict of interest.

## Publisher's Note

All claims expressed in this article are solely those of the authors and do not necessarily represent those of their affiliated organizations, or those of the publisher, the editors and the reviewers. Any product that may be evaluated in this article, or claim that may be made by its manufacturer, is not guaranteed or endorsed by the publisher.

## References

[1] Page K Marwick TH Lee R Grenfell R Abhayaratna WP Aggarwal A. A systematic approach to chronic heart failure care: a consensus statement. Med J Aust. (2014) 201:146–50. 10.5694/mja14.00032 25128948

[2] Maggioni AP Dahlström U Filippatos G Chioncel O Leiro MC Drozdz J. EURObservational research programme: regional differences and 1-year follow-up results of the Heart Failure Pilot Survey (ESC-HF Pilot). Eur J Heart Fail. (2013) 15:808–17. 10.1093/eurjhf/hft050 23537547

[3] Ziaeian B Fonarow GC. Epidemiology and aetiology of heart failure. Nat Rev Cardiol. (2016) 13:368–78. 10.1038/nrcardio.2016.25 26935038 PMC4868779

[4] Atherton JJ Sindone A De Pasquale CG Driscoll A MacDonald PS Hopper I. National heart foundation of Australia and cardiac society of Australia and New Zealand: guidelines for the prevention, detection, and management of heart failure in Australia 2018. Heart Lung Circ. (2018) 27:1123–208. 10.1016/j.hlc.2018.06.1042 30077227

[5] McDonagh TA Metra M Adamo M Gardner RS Baumbach A Böhm M. 2021 ESC guidelines for the diagnosis and treatment of acute and chronic heart failure. Eur Heart J. (2021) 42:3599–726. 10.1093/eurheartj/ehab368 34447992

[6] Jaarsma T Hill L Bayes-Genis A La Rocca HPB Castiello T Celutkiene J. Self-care of heart failure patients: practical management recommendations from the heart failure association of the European society of cardiology. Eur J Heart Fail. (2021) 23:157–74. 10.1002/ejhf.2008 32945600 PMC8048442

[7] Jonkman NH Westland H Groenwold RH Ågren S Atienza F Blue L. Do self-management interventions work in patients with heart failure? An individual patient data meta-analysis. Circulation. (2016) 133:1189–98. 10.1161/CIRCULATIONAHA.115.018006 26873943 PMC5180429

[8] Bui AL Fonarow GC. Home monitoring for heart failure management. J Am Coll Cardiol. (2012) 59:97–104. 10.1016/j.jacc.2011.09.044 22222071 PMC3254025

[9] Segan L Nanayakkara S Mak V Kaye D. Enhancing self-care strategies in heart failure through patient-reported outcome measures. Intern Med J. (2018) 48:995–8. 10.1111/imj.13977 30133978

[10] Farwati M Riaz H Tang WW. Digital health applications in heart failure: a critical appraisal of literature. Curr Treat Options Cardiovasc Med. (2021) 23:1–11. 10.1007/s11936-020-00881-3 33488049 PMC7812033

[11] Kitsiou S Vatani H Paré G Gerber BS Buchholz SW Kansal MM. Effectiveness of mobile health technology interventions for patients with heart failure: systematic review and meta-analysis. Can J Cardiol. (2021) 37:1248–59. 10.1016/j.cjca.2021.02.015 33667616

[12] Islam SMS Farmer AJ Bobrow K Maddison R Whittaker R Dale LAP. Mobile phone text-messaging interventions aimed to prevent cardiovascular diseases (Text2PreventCVD): systematic review and individual patient data meta-analysis. Open Heart. (2019) 6:e001017. 10.1136/openhrt-2019-001017 31673381 PMC6802999

[13] Lin MH Yuan WL Huang TC Zhang HF Mai JT Wang JF. Clinical effectiveness of telemedicine for chronic heart failure: a systematic review and meta-analysis. J Investig Med. (2017) 65:899–911. 10.1136/jim-2016-000199 28330835

[14] Birnbaum F Lewis D Rosen RK Ranney ML. Patient engagement and the design of digital health. Acad Emerg Med. (2015) 22:754–6. 10.1111/acem.12692 25997375 PMC4674428

[15] Burrows A Meller B Craddock I Hyland F Gooberman-Hill R. User involvement in digital health: Working together to design smart home health technology. Health Expectations. (2019) 22:65–73. 10.1111/hex.12831 30289590 PMC6351410

[16] Shah SGS Robinson I. User involvement in healthcare technology development and assessment: structured literature review. Int J Health Care Qual Assur. (2006) 19:500–15. 10.1108/09526860610687619 17100220

[17] Dening J George ES Ball K Islam SMS. User-centered development of a digitally-delivered dietary intervention for adults with type 2 diabetes: the T2Diet study. Internet Interv. (2022) 28:100505. 10.1016/j.invent.2022.100505 35242592 PMC8861390

[18] Amiribesheli M Benmansour A Bouchachia A. A review of smart homes in healthcare. J Ambient Intell Humaniz Comput. (2015) 6:495–517. 10.1007/s12652-015-0270-2

[19] Deen MJ. Information and communications technologies for elderly ubiquitous healthcare in a smart home. Pers Ubiquitous Comput. (2015) 19:573–99. 10.1007/s00779-015-0856-x

[20] Islam SMS Halooq A Dening J Uddin R Laranjo L Chow C. Healthcare Providers' Perspectives on Using Smart Home Systems to Improve Self-Management and Care in People with Heart Failure: A Qualitative Study. Available online at: https://papers.ssrn.com/sol3/papers.cfm?abstract_id=3992283 (accessed June 17, 2022). 10.1016/j.ijmedinf.2022.104837 36126353

[21] Muse ED Barrett PM Steinhubl SR Topol EJ. Towards a smart medical home. Lancet. (2017) 389:358. 10.1016/S0140-6736(17)30154-X 28137686 PMC5604864

[22] Helal A Cook DJ Schmalz M. Smart home-based health platform for behavioral monitoring and alteration of diabetes patients. J Diabetes Sci Technol. (2009) 3:141–8. 10.1177/193229680900300115 20046657 PMC2769843

[23] Moses JC Adibi S Angelova M Islam SMS. Smart home technology solutions for cardiovascular diseases: a systematic review. Appl Syst Innov. (2022) 5:51. 10.3390/asi5030051

[24] Mshali H Lemlouma T Moloney M Magoni D. A survey on health monitoring systems for health smart homes. Int J Ind Ergon. (2018) 66:26–56. 10.1016/j.ergon.2018.02.002

[25] Jünger S Payne SA Brine J Radbruch L Brearley SG. Guidance on Conducting and REporting DElphi Studies (CREDES) in palliative care: recommendations based on a methodological systematic review. Palliat Med. (2017) 31:684–706. 10.1177/0269216317690685 28190381

[26] Black N Murphy M Lamping D McKee M Sanderson C Askham J. Consensus development methods: a review of best practice in creating clinical guidelines. J Health Serv Res Policy. (1999) 4:236–48. 10.1177/135581969900400410 10623041

[27] Jones J Hunter D. Consensus methods for medical and health services research. BMJ. (1995) 311:376. 10.1136/bmj.311.7001.376 7640549 PMC2550437

[28] Pandor A Kaltenthaler E Martyn-St James M Wong R Cooper K Dimairo M. Delphi consensus reached to produce a decision tool for SelecTing Approaches for Rapid Reviews (STARR). J Clin Epidemiol. (2019) 114:22–9. 10.1016/j.jclinepi.2019.06.005 31185276

[29] Guyatt GH Oxman AD Kunz R Vist GE Falck-Ytter Y Schünemann HJ. What is “quality of evidence” and why is it important to clinicians? BMJ. (2008) 336:995–8. 10.1136/bmj.39490.551019.BE 18456631 PMC2364804

[30] Yancy CW Jessup M Bozkurt B Butler J Casey DE Jr Colvin MM. 2017 ACC/AHA/HFSA focused update of the 2013 ACCF/AHA guideline for the management of heart failure: a report of the American college of cardiology/American heart association task force on clinical practice guidelines and the heart failure society of America. J Am Coll Cardiol. (2017) 70:776–803. 10.1016/j.jacc.2017.04.025 28461007

[31] Real J Cowles E Wierzbicki AS. Chronic heart failure in adults: summary of updated NICE guidance. BMJ. (2018) 362:k3646. 10.1136/bmj.k3646 30249604

[32] Baldwin CE Phillips AC Edney SM Lewis LK. Recommendations for older adults' physical activity and sedentary behaviour during hospitalisation for an acute medical illness: an international Delphi study. Int J Behav Nutr Phys Act. (2020) 17:1–17. 10.1186/s12966-020-00970-3 32450879 PMC7249667

[33] Sucala M Ezeanochie NP Cole-Lewis H Turgiss J. An iterative, interdisciplinary, collaborative framework for developing and evaluating digital behavior change interventions. Transl Behav Med. (2019) 10:1538–48. 10.1093/tbm/ibz109 31328775 PMC7796712

[34] Ashton CM Kuykendall DH Johnson ML Wray NP Carr MJ Slater CH. A method of developing and weighting explicit process of care criteria for quality assessment. Med Care. (1994) 32:755–70. 10.1097/00005650-199408000-00001 8057693

[35] Heiko A. Consensus measurement in Delphi studies: review and implications for future quality assurance. Technol Forecast Soc Change. (2012) 79:1525–36. 10.1016/j.techfore.2012.04.013

[36] McMillan SS King M Tully MP. How to use the nominal group and Delphi techniques. Int J Clin Pharm. (2016) 38:655–62. 10.1007/s11096-016-0257-x 26846316 PMC4909789

[37] Belton I MacDonald A Wright G. Hamlin I. Improving the practical application of the Delphi method in group-based judgment: a six-step prescription for a well-founded and defensible process. Technol Forecast Soc Change. (2019) 147:72–82. 10.1016/j.techfore.2019.07.002

[38] De Villiers MR De Villiers PJ Kent AP. The Delphi technique in health sciences education research. Med Teach. (2005) 27:639–43. 10.1080/13611260500069947 16332558

[39] Abdalrada A Abawajy J Al-Quraishi T Islam S. Machine learning models for prediction of co-occurrence of diabetes and cardiovascular diseases: a retrospective cohort study. J Diabetes Metab Disord. (2022) 21:251–61. 10.1007/s40200-021-00968-z 35673486 PMC9167176

[40] Abdalrada AS Abawajy J Al-Quraishi T Islam SMS. Prediction of cardiac autonomic neuropathy using a machine learning model in patients with diabetes. Ther Adv Endocrinol Metab. (2022) 13:20420188221086693. 10.1177/20420188221086693 35341207 PMC8943459

[41] Islam S Talukder A Awal M Siddiqui M Ahamad M Ahammed B. Machine learning approaches for predicting hypertension and its associated factors using population-level data from three South Asian countries. Front Cardiovasc Med. (2022) 9:839379. 10.3389/fcvm.2022.839379 35433854 PMC9008259

[42] Islam S Cartledge S Karmakar C Rawstorn J Fraser S Chow C. Validation and acceptability of a cuffless wrist-worn wearable blood pressure monitoring device among users and healthcare professionals: a mixed-method study. JMIR mHealth uHealth. (2019) 7:e14706. 10.2196/14706 31628788 PMC6827985

[43] Islam SMS Chow CK Daryabeygikhotbehsara R Subedi N Rawstorn J Tegegne T. Wearable cuffless blood pressure monitoring devices: a systematic review and meta-analysis. Eur Heart J-Digital Health. (2022). 10.1093/ehjdh/ztac021 [Epub ahead of print]. PMC9708022 36713001

[44] Islam SMS Khosravi A. The need for a prediction model assessment framework. Lancet Glob Health. (2021) 9:e404. 10.1016/S2214-109X(21)00022-X 33581049 PMC7906664

[45] Zaman SB Khan RK Evans RG Thrift AG Maddison R Islam SMS. Exploring barriers to and enablers of the adoption of information and communication technology for the care of older adults with chronic diseases: scoping review. JMIR Aging. (2022) 5:e25251. 10.2196/25251 34994695 PMC8783284

